# Habit Formation to Enhance Implementation of Evidence-based Practice

**DOI:** 10.1007/s10488-026-01519-5

**Published:** 2026-08-03

**Authors:** Zabin Patel-Syed, Kelli Scott, Sarah Helseth, Sara Becker

**Affiliations:** 1 Institute for Public Health and Medicine, Center for Dissemination and Implementation Science, Northwestern University Feinberg School of Medicine, Chicago, USA; 2 Department of Psychiatry and Behavioral Sciences, Northwestern University Feinberg School of Medicine, Chicago, USA

**Keywords:** Habit, Implementation science, Evidence-based practice, Healthcare, Behavior change

## Abstract

Healthcare professionals often report strong intentions to use evidence-based practices to improve patient care, but intention does not always translate into action. Habits, automatic behaviors generated through repeated cue-behavior associations, may help address the intention-behavior gap and enhance adoption of evidence-based practices. As habits form, behavior control shifts from being internally guided to being triggered by external context cues, which can support making the use of evidence-based practice more routine. Despite calls to integrate habit formation in implementation science, habits have not yet been fully leveraged to select and specify implementation strategies. We argue that habits can be explicitly planned for with targeted cues, behavior routines, and rewards to support healthcare professionals in adopting evidence-based practices. We outline core elements of habit formation and provide case examples to illustrate how habit techniques can be used to catalyze implementation in healthcare.

Implementation science has emerged in response to a well-documented “know-do gap” in healthcare (Bauer et al., 2015). Importantly, implementation requires behavior change among healthcare professionals who will ultimately deliver an evidence-based practice (EBP; Nilsen et al., 2022; Presseau et al., 2019). Thus, theories of behavior change are critical to understanding drivers of effective implementation (Michie et al., 2009, 2013).

Implementation studies draw largely on social cognitive theories of behavior change to explain why healthcare professionals do—or do not—adopt an EBP (Grimshaw et al., 2012). Social cognitive theories, such as the Theory of Planned Behavior (TPB; Ajzen, 1991), are predicated on the assumption that *intention* to engage in a behavior predicts action. TPB posits that intentions are goal-directed and influenced by: *attitudes towards the behavior* (whether an EBP is positively or negatively valued), *subjective norms* (social pressures to use an EBP), and *perceived control over the behavior* (beliefs about one’s ability to use an EBP). Consistent with social cognitive models, implementation strategies often explicitly target intentions to use EBPs as an individual-level determinant of uptake (Powell et al., 2015).

Intention is a well-established predictor of behavior change but can also fail to translate into action (Sheeran & Webb, 2016). The *intention-behavior gap* has been studied widely in clinical behaviors including prescribing, providing referrals, and adhering to clinical guidelines. In 2008, Godin and colleagues systematically reviewed 72 studies of social cognitive theories and found that intention predicted only 31% of the variance in behavior among healthcare professionals. This suggests that targeting decision-making processes that influence behavioral intentions is necessary, but insufficient to change healthcare professional behavior. As Potthoff and colleagues (2019) asserted, there is a clear need to move beyond deliberate decision-making and consider *implicit*, or automatic, processes in implementation.

We argue that habits, automatic behaviors generated in response to external context cues through repetition and reinforcement, represent an implicit process with strong potential to increase the adoption of EBPs (Gardner & Lally, 2018; Verplanken, 2018). Habits are actions that have come under the control of an automatic cognitive process, which can override intentions when motivation is low (e.g., feeling tired at the end of a busy workday). As such, habits complement extant implementation mechanisms focused on deliberate decision-making by offloading cognitive control to environmental cues that promote automatic behaviors. Nilsen (2015) and Potthoff et al. (2017) argued that habits have not been sufficiently explored in implementation efforts to change healthcare professionals’ behavior; a decade later, we believe that these assertions still ring true. Habits are a behavior change mechanism that can help with implementation strategy selection and specification. We outline three core elements that promote habit formation and provide case examples to demonstrate how habits can be employed to advance implementation science and practice.

## Habits: A Conceptual Overview

Habits are characterized by *automaticity*, a cognitive short-cut in which an individual does not actively deliberate on the consequences of engaging in a behavior (Wood & Neal, 2007). Lally and Gardner (2013) proposed a conceptual framework that organizes habit formation into four phases: deciding to act, acting on that decision, performing the behavior in the presence of contextual cues, and developing an implicit cue-behavior association. As habits develop, behavioral control gradually shifts from deliberate decision-making to being triggered by cues in the environment (Gardner et al., 2020; Gardner & Lally, 2018).

A meta-analysis of nine studies with 1,975 healthcare professionals found a medium effect size (*r* = .35) for the relationship between habits and everyday clinical behaviors (Potthoff et al., 2019); these data demonstrate that habits are a significant predictor of healthcare professionals’ behavior and a key driver of implementation. Leveraging habits to promote EBP adoption requires understanding how they are developed and maintained. The “habit loop” is a three-part process that typically occurs during the third and four phases of habit formation. It involves defining a target behavior and identifying three elements: (1) cue, (2) behavior routine, and (3) reward (Carden & Wood, 2018; Wood & Neal, 2016). Table 1 presents three case examples of implementation trials that harnessed these elements to promote health professional behavior change. We describe each element below and use case examples to demonstrate the clinical utility of employing habit techniques.

## Cue

Habits are prompted by cues. Repeating a behavior in the presence of specific cues over time strengthens the cue-behavior association, making behavior more reliant on external triggers and less on conscious decision-making (Verplanken, 2018). Strategies to optimize the use of context cues include: (1) assessing and observing organizational workflow patterns that may precede habit initiation; (2) identifying cues that can be feasibly used, within organizational constraints, to trigger habit development; and (3) modifying the physical environment to make cues clear, easily understandable, and obvious (Gardner, 2015). Cues can be internal (e.g., thoughts, physical sensations), external (e.g., visuals, checklists, alarms), event-based (e.g., prior actions), or time-based (e.g., time of day). Highly salient, frequent, and consistently encountered cues can accelerate habit formation (Wood & Neal, 2007). Identifying and tailoring cue selection to a specific healthcare context is critical to ensure acceptability and increase the salience of a target behavior (Judah et al., 2013; Mohideen et al., 2023). Little is known about which cues effectively promote habits in high demand clinical settings, or their acceptability to healthcare professionals, representing an important direction for future research.

### Cues: Case Example #1

IAMSBIRT (Implementing Alcohol Misuse Screening, Brief Intervention, and Referral to Treatment) was a hybrid type 3 effectiveness-implementation trial evaluating a multi-level strategy to promote the implementation of Screening, Brief Intervention, and Referral to Treatment (SBIRT) across 10 pediatric trauma centers (Mello et al., 2024). Both visual and structured cues were used to promote the Screening component of SBIRT. Screening was conducted with the S2BI, a validated screening tool, and delivered by nurses. Visual cues were prominently displayed in areas where nurses gathered, to generate “buzz” about the implementation initiative and get nurses regularly thinking about screening. Visual posters describing the initiative with the tagline, “I S2BI, ask me why!” were posted in the nurses’ staff room and at the nurses’ station, and most critically, all nursing staff were given badge holders with the initiative tagline, which they were encouraged to wear throughout the workday. Structural cues included embedding the S2BI into the standard intake form within the electronic medical record (EMR). Within the intake form, open-ended fields were removed and response options were added that mapped directly onto the S2BI. These structural cues made it more effortful for nurses to ask open ended or unvalidated questions, while reducing the effort required to ask the S2BI questions. Results of this trial suggested that the cues and other implementation strategies used were successful: screening rates increased from 25.2% to 47.7% at sites from the pre- to post-implementation phase (Mello et al., 2024).

## Behavior Routine

Exposure to cues prompts a behavior response, which becomes automated through repetition (Wood & Neal, 2016). Routines provide a structured sequence of actions that promote habits through repetition and predictability, reducing cognitive load (Lally & Gardner, 2013). Establishing behavior routines aligned with the regular use of EBPs in healthcare is critical to make behavior change more automatic over time. Taking an incremental, “small changes” approach to EBP implementation may reduce fidelity to delivery but setting overly ambitious goals may lead to discouragement and disengagement (Verplanken, 2018). Striking a balance between realistic goals and effective routines is essential for long-term behavior change. Other best practices for habit formation include breaking the target behavior into manageable steps and creating a system for healthcare professionals to monitor their progress (Lally & Gardner, 2013).

### Behavior Routine: Case Example #2

The Brown University Alcohol Research Center on HIV (ARCH) was a center with three major research components, one of which aimed to promote the implementation of SBIRT targeting risky alcohol use across a national non-governmental HIV service organization in South Africa (Kuo et al., 2021). The primary implementation strategy was a train-the-trainer model that relied heavily on generating behavior routines. Lay counselors delivering SBIRT were provided a tablet with SBIRT questions pre-programmed into the intake form, to make screening part of the standard workflow. Providers were also given an SBIRT self-reflection form and encouraged to complete it after each intake. The self-reflection form was designed to break up the SBIRT process into discrete, brief steps, while completion of the form after each intake was intended to foster a convenient, regular system for counselors to track their progress. Quantitative data revealed that 55,751 individuals were screened in the first year of SBIRT implementation, and qualitative data indicated that providers appreciated the emphasis on self-assessment and routine tracking (DiClemente-Bosco et al., 2025).

## Rewards

A habit is strengthened by rewards (Gardner & Lally, 2018). Tangible incentives are widely used in implementation because rewards provide positive reinforcement, immediate confirmation that a behavior was performed correctly, and increase the likelihood of future behavior repetition (Carden & Wood, 2018). Best practices in using rewards include (Lally & Gardner, 2013): (1) identifying acceptable and meaningful rewards that align with an organization’s needs, values, and resources; (2) ensuring that rewards are provided immediately, consistently, and tied to the of an EBP; and (3) developing a reward schedule.

Several attributes of rewards influence habit formation. Rewards can be monetary (e.g., financial incentives) or non-monetary (e.g., public praise; Neal et al., 2006; Verplanken, 2018). Additionally, rewards can be provided on a consistent schedule (e.g., after every instance of delivering an EBP) or intermittently (e.g., through periodic feedback, incentives, or recognition of delivery). As EBP delivery becomes more integrated into the clinical workflow, gradually shifting from a consistent to intermittent schedule may reduce reliance on ongoing external reinforcement (Lally & Gardner, 2013). Given that habit formation among healthcare professionals requires behavior change within busy, multi-layered systems, this may look like celebrating staff achievements at each team meeting, embedding positive performance feedback into team huddles, or using verbal praise to reinforce EBP delivery. Rewards are key to motivating healthcare professionals and promoting sustainable change (Neal et al., 2006).

### Rewards: Case Example #3

Project MIMIC was a hybrid type 3 effectiveness-implementation trial that tested two multi-level implementation strategies to promote the implementation of contingency management (CM) in opioid treatment programs (Becker et al., 2021). The two strategies were the standard multi-level strategy used by the Addiction Technology Transfer Centers and an enhanced strategy that layered in facilitation, to promote internal motivation, and incentivization, to promote external motivation. Providers randomized to the enhanced ATTC strategy could earn $50 for every month they meet a skills target, and $200 for every patient that met an attendance target. Over the course of the trial, providers randomized to the enhanced ATTC condition earned $18,450 in financial incentives. Results indicated that providers receiving the enhanced ATTC strategy had about two to four times greater odds of adopting CM, meeting the CM exposure benchmark, and meeting the CM skills benchmark (Becker et al., 2025).

## Conclusions

Habits may be particularly effective in helping health professionals make the delivery of evidence-based practices routine. This can help ensure that behavior change is sustained even after the withdrawal of active implementation supports. In other words, habits have the potential to protect against the “voltage drop” (Chambers et al., 2013) often observed in implementation trials. Several authors have emphasized the importance of integrating habits into implementation (Gardner et al., 2020; Nilsen et al., 2012), making this area ripe for applied study with notable considerations. First, future work should test whether using Lally and Gardner’s (2019) conceptual framework to guide selection, specification, and bundling of behavior change techniques improves the effectiveness of implementation strategies. A direct comparison would help identify active ingredients of implementation strategies and provide empirical evidence on the added value of incorporating habit techniques to target behavioral barriers (Lewis et al., 2022). Second, future research should explore how long it takes healthcare professionals to develop habits related to EBP delivery and the most appropriate methods (e.g., self-report, direct observation, rapid ethnography) for measuring habit formation in busy clinical settings. Researchers need pragmatic guidance on appropriate timeframes to inform evaluation plans that specify *when* and *how* to assess habit formation. Finally, deimplementing inappropriate health interventions is critical for minimizing patient harm and promoting public health (Norton & Chambers, 2020). Investigating habits in healthcare also holds potential for addressing harmful clinical behaviors, such as removing cues that prompt use of ineffective practices and encourage habit substitution, thereby reducing negative care consequences.

In conclusion, we echo previous calls to integrate habits more fully into implementation research and aim to inspire researchers to design and test habit interventions to facilitate behavior change among healthcare providers. We also extend prior research by providing a set of illustrative case examples that demonstrate how to apply these interventions in routine health care settings to promote the uptake of evidence-based interventions.

## Figures and Tables

**Table 1:** Promoting Habit Formation: Illustrative Implementation Trial Case Examples

Study overview and habit component	Case example #1: IAMSBIRT	Case example #2: ARCH	Case example #3: MIMIC
Intervention being implemented	Screening, brief intervention, and referral to treatment for risky alcohol use (SBIRT)	Screening, brief intervention, and referral to treatment for risky alcohol use	Contingency management, an intervention in which patients earn prizes for meeting treatment goals
Setting	Pediatric trauma centers	HIV service settings	Opioid treatment programs
Individuals implementing the intervention	Nurses and social workers	Lay counselors and community health workers	Substance use counselors
Cues	• *Visual cues* – posters with SBIRT tag line (“IS2BI, ask me why!” displayed prominently in nursing break rooms, badges provided to nurses to wear daily, session note pads given to social workers • *Structural cues* – screening was programmed into the medical record requiring staff to opt out of completion	• *Structural cues* – screening was programmed into the routine intake assessment, which was administered via tablets; fields to record open ended responses were removed to make open-ended questions more difficult to record	• *Visual cues* – prize cabinets were decorated and placed in prominent areas within each treatment program, prize menus were displayed in counseling rooms to increase visual salience
Behavior Routine	• *Electronic Medical Record (EMR) modifications* – structural cues were built in the EMR to facilitate behavior repetition, including automatic alert to trigger a social work consult be ordered for adolescents with substance use	• *Integration into the standard intake assessment form* – the screening tool was embedded within the organizations’ standard intake assessment form via a tablet, and a full risk algorithm was built into the tablet to fully automate the SBIRT workflow • *Checklist completion* – Community health workers were required to complete a checklist at the end of each screening encounter to promote routine tracking	• *Use of a standardized contingency management tracker* – providers were trained to use a contingency management tracking system that provided structural cues and reminders for how to deliver with fidelity and to track each patient’s number of prize draws each week
Rewards	• *Non-monetary reinforcement* – pediatric trauma center staff were rewarded for using SBIRT through ongoing feedback and social reinforcement delivered on the trauma unit by leadership and during monthly coaching calls with the research team	• *Non-monetary reinforcement* – ARCH employed three non-monetary rewards: 1) a community health worker designated as a “master trainer” was used to provide ongoing feedback and reinforcement 2)provision of positive reinforcement built into the tablet-based assessment 3)frequent provision of positive reinforcement from research staff and program administrators; celebrations for attaining milestones (e.g., screening 1000 patients)	• *Non-monetary reinforcement* – opioid treatment program staff received verbal praise during external facilitation designed to promote competence, confidence, and internal motivation • *Monetary reinforcement* - Project MIMIC explicitly tested if financial incentives accelerate adoption of contingency management so opioid treatment program staff received $50 for every month they met a skills target on a contingency management session and $200 for every patient that received at least 10 contingency management sessions

## Data Availability

No datasets were generated or analysed during the current study.
